# Mechanism of differential expression of β-glucosidase genes in functional microbial communities in response to carbon catabolite repression

**DOI:** 10.1186/s13068-021-02101-x

**Published:** 2022-01-12

**Authors:** Xinyue Zhang, Xiehui Chen, Shanshan Li, Ayodeji Bello, Jiawen Liu, Liyuan Gao, Zhihua Fan, Shouzhi Wang, Libo Liu, Bo Ma, Hongtao Li

**Affiliations:** 1 College of Resources and Environmental Sciences, Northeast Agricultural University, Harbin, 150030 China; 2 College of Animal Science and Technology, Northeast Agricultural University, Harbin, 150030 China; 3 School of Animal Medicine, Northeast Agricultural University, Harbin, 150030 China; 4 Northeastern Science Inspection Station, China Ministry of Agriculture Key Laboratory of Animal Pathogen Biology, Harbin, 150030 China; 5 College of Food Science, Northeast Agricultural University, Harbin, 150030 China

**Keywords:** β-Glucosidase, Differential expression, Functional microbial community, Carbon catabolite repression (CCR), Compost

## Abstract

**Background:**

β-Glucosidase is the rate-limiting enzyme of cellulose degradation. It has been stipulated and established that β-glucosidase-producing microbial communities differentially regulate the expression of glucose/non-glucose tolerant β-glucosidase genes. However, it is still unknown if this differential expression of functional microbial community happens accidentally or as a general regulatory mechanism, and of what biological significance it has. To investigate the composition and function of microbial communities and how they respond to different carbon metabolism pressures and the transcriptional regulation of functional genes, the different carbon metabolism pressure was constructed by setting up the static chamber during composting.

**Results:**

The composition and function of functional microbial communities demonstrated different behaviors under the carbon metabolism pressure. Functional microbial community up-regulated glucose tolerant β-glucosidase genes expression to maintain the carbon metabolism rate by enhancing the transglycosylation activity of β-glucosidase to compensate for the decrease of hydrolysis activity under carbon catabolite repression (CCR). *Micrococcales* play a vital role in the resistance of functional microbial community under CCR. The transcription regulation of GH1 family β-glucosidase genes from Proteobacteria showed more obvious inhibition than other phyla under CCR.

**Conclusion:**

Microbial functional communities differentially regulate the expression of glucose/non-glucose tolerant β-glucosidase genes under CCR, which is a general regulatory mechanism, not accidental. Furthermore, the differentially expressed β-glucosidase gene exhibited species characteristics at the phylogenetic level.

**Supplementary Information:**

The online version contains supplementary material available at 10.1186/s13068-021-02101-x.

## Background

Cellulose degradation is the largest renewable carbon source in the Earth’s biosphere and it is a significant process in the biogeochemical cycling of carbon. Endoglucanase (E.C.3.2.1.4), exoglucanase (E.C.3.2.1.91) and β-glucosidase (E.C.3.2.1.21) act synergistically for the complete hydrolysis of cellulose [1]. Cellulase synthesis by microorganisms is regulated by the induction-repression system [2]. Metabolic repression (product repression) is the primary regulatory mechanism for microorganisms to control their enzyme production [3]. β-Glucosidase completes the final step of hydrolysis by converting the cellobiose (an intermediate product of cellulose hydrolysis) and some oligosaccharides to glucose, alleviating the negative feedback regulation of exoglucanase and endoglucanase biosynthesis caused by elevated cellobiose levels [4, 5]. However, in some cases, glucose produced by β-glucosidase accumulates and inhibits cellulase production [6, 7]. In addition, β-glucosidase is known to play an important role in regulating the synthesis of other enzyme components and is recognized as a rate-limiting enzyme in the microbial degradation of cellulose [8–10]. Twenty transcription factors from *Penicillium oxalicum* have been identified to play a role in the activation or repression of cellulase synthesis [11]. The study refined the transcriptional-regulatory network as a “seesaw model” in which the coordinated regulation of cellulolytic genes is established by counteracting activators (ClrB and XlnR) and repressors (CreA and AmyR) which affected by glucose and cellobiose, respectively. The key of “seesaw model” directly focuses on the metabolic process of β-glucosidase.

Berlemont and Martini [12] reported that β-glucosidase genes are present in almost all bacterial phyla, confirmed by Pathan et al. [13]. Unlike endoglucanase and exoglucanase, β-glucosidase can be expressed by potential degraders and potential opportunists [12]. These facts revealed that a wide phylogenetic diversity of microorganisms is likely to be involved in the final step of enzymatic cellulose hydrolysis. Several sets of degenerate primers have been designed to analyze β-glucosidase gene diversity from different environmental niches [13–17]. However, few attempts have been made to explore microbial diversity based on their metabolic or functional potential.

High glucose inhibits the activity and expression of β-glucosidase [18, 19]. However, some β-glucosidases (BGL) are uninhibited by high glucose concentration in recent decades [20–22]. The glucose tolerant β-glucosidases in biomass utilization have received more attention, but the regulatory mechanism and biological significance of functional microbes encoding and expressing glucose-tolerant β-glucosidase in the natural environment is still unclear.

Previous study revealed that glucose/non-glucose tolerant β-glucosidases had opposite transcriptional regulation patterns in the cooling phase of natural compost (CCR occurred) [7]. In addition, the hypothesis that “β-glucosidase-producing microbial communities differentially regulate expression of glucose/non-glucose-tolerant β-glucosidase genes to adapt to changes in cellulose degradation” was verified. The phenomenon of β-glucosidase genes differential expression in some cellulose decomposing microbes such as *Trichoderma reesei*, *A. oryzae*, *Clostridium thermocellum*, and *Aspergillus terreus* have been documented [20, 23–28]. However, the “when, why, and how” related to glucose-tolerant β-glucosidase regulation by β-glucosidase-producing microbial communities in a natural environment remains to be elucidated. Answers to these questions are critical for understanding how microbial populations interact with substrates and products to drive fundamental ecological processes of cellulose degradation. In this study, the different carbon metabolism conditions were constructed by adding glucose, cellobiose and d-gluconate-1.5-lactone in the early thermophilic phase (T1 phase), the later thermophilic phase (T2 phase) and the cooling phase (C phase) of composting. Metagenome, metatranscriptome, and DNA library targeting GH1 family β-glucosidase were used to systematically analyze the microbial community composition and function and differential expression of glucose/non-glucose tolerant β-glucosidase genes under CCR effect.

## Results

### *The activities of key enzymes*, *content of products during cellulose degradation*

Temperature profiles followed the typical dynamics of the aerobic composting process, including the mesophilic phase (before day 3), thermophilic phase (days 3–28), and cooling phase (days 28–43) (Additional file 1: Fig. S1). Cellulose is a recalcitrant carbon source and its utilization by microorganisms has regular characteristics at different stages in composting. At the mesophilic phase and early thermophilic phases, microbes preferentially make use of simple carbon sources, such as starch and polysaccharide. Cellulose was highly degraded and utilized by cellulolytic microbes during later thermophilic and cooling phases.

**Fig. 1:**
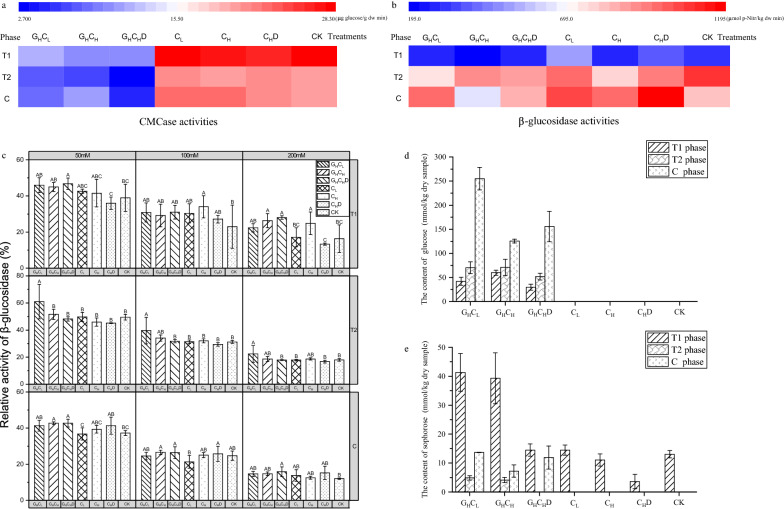
Activities of key enzymes, content of products during cellulose degradation at T1, T2 and C phases. **a** Activity of CMCase. **b** Activity of β-glucosidase. **c** Relative activities of β-glucosidase under different concentration of glucose (50 mM, 100 mM and 200 mM). **d** Content of glucose. **e** Content of sophorose

The CMCase activity in different treatments is shown in Fig. 1. CMCase activity of all treatments with high concentration of glucose (G_H_C_L_, G_H_C_H_, and G_H_C_H_D) was significantly lower than that of other treatments, which was consistent with Nitta et al. [29] observation that high glucose inhibited cellulase output. This phenomenon was observed at the T1, T2, and C phases, suggesting that the inhibition was unaffected by time gradient or different phases of cellulose degradation.

Although CMCase and β-glucosidase act synergistically during the degradation of cellulose, the peak activity value of β-glucosidase was delayed compared to that of CMCase activity (Fig. 1a, b). This result was also observed in the DGGE profile of Li’s [30] study. The β-glucosidase activity of all samples in the T1 phase (190–520 μmol p-Nitr/kg dw min) was obviously lower than the T2 and C phases (780–1200 μmol p-Nitr/kg dw min) (Fig. 1b). This result indicated that the cellulose degradation in the T1 phase was limited to the step that β-glucosidase hydrolyzes cellobiose to glucose as compared with the T2 and C phases. Interestingly, unlike the change of CMCase activity, the β-glucosidase activity was not inhibited by high glucose. To further explore the glucose tolerance characteristics of β-glucosidase in each treatment. The relative activity of β-glucosidase under gradient glucose concentration (50, 100 and 200 mM) was determined (Fig. 1c). Results showed that the relative activity of β-glucosidase decreased with the increase in glucose concentration. Notably, the β-glucosidase of treatment G_H_C_L_ was more tolerant than other treatments at T2 phase, indicating that the functional microbial communities regulated produce more glucose-tolerant β-glucosidase. This was also reflected in the ratio of glucose tolerant β-glucosidase genes which peaked in G_H_C_L_ treatment from functional genes clone library at T2 phase (discussion as below).

Glucose, which is the end products of cellulose degradation, can only be detected in all treatments with high glucose at T1, T2 and C phases (Fig. 1d). The glucose content reached the peak at the C phase and lowest at the T1 phase (Fig. 1d). These results showed that the uptake and metabolism of glucose by microorganisms are faster at the T1 phase, and the metabolism of microorganisms is reduced at the cooling phase of compost compared with the thermophilic phase.

Sophorose, a natural inducer of cellulase and the transglycosylation product of β-glucosidase [31], was observed in all treatments with high glucose at T1, T2 and C phases except treatment G_H_C_H_D at T2 phase (Fig. 1e). However, sophorose was also detected in treatment without glucose at the T1 phase (Fig. 1e).

### The composition and co-occurrence network of functional microbial community

**Fig. 2:**
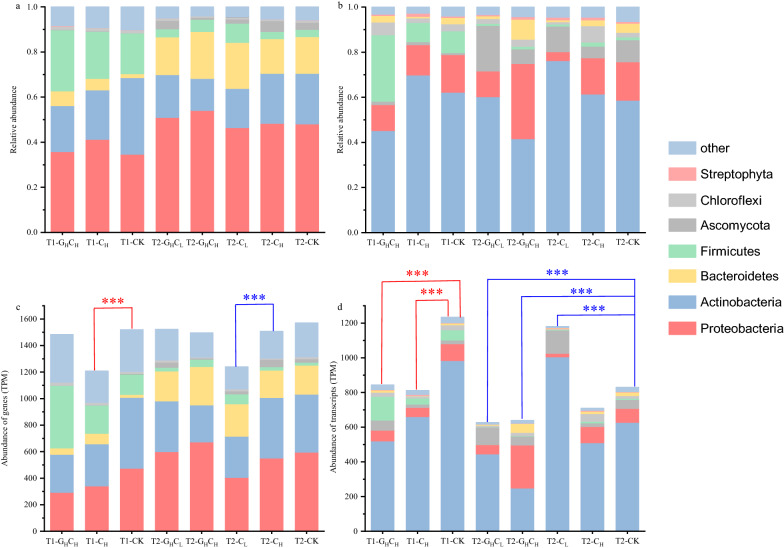
Relative taxonomic distribution of metagenome and metatranscriptome. **a** Total gene at the phylum level in metagenome. **b** Total gene at the phylum level in metatranscriptome. **c** Cellulolytic gene at the phylum level in metagenome. **d** Cellulolytic gene at the phylum level in metatranscriptome. Signifcant diferences in read abundances between the treatments and control composting phases are indicated by stars (*P* < 0.05)

The microbial community composition and function were determined by integrated metatranscriptomic and metagenomic analyses. For the whole microbial community, at the DNA level, four phylum-level groups were observed to be predominant: *Proteobacteria*, *Actinobacteria*, *Bacteroidetes* and *Firmicutes* (Fig. 2a). Notably, these four phyla were also reported as the dominant phyla in other lignocellulosic composts [32, 33]. The microbial community composition in the T1 and T2 phases showed obvious differences. *Firmicutes*, for instance, have a higher relative abundance in the T1 phase than in the T2 phase, and *Bacteroidetes* have a higher relative abundance in the T2 phase than in the T1 phase. The result of cluster analysis showed that the effect of the composting phase plays an important role in the formation of microbial community composition (Additional file 1: Fig. S3a). However, at transcript level, *Actinobacteria* was the only dominant phylum (Fig. 2b), indicating that this phylum plays an essential role in cellulose degradation during the composting process [34]. Furthermore, cluster analysis also showed that the functional microbial community was sensitive to external carbon source addition, especially in G_H_C_H_ treatment with the highest carbon metabolism pressure (Additional file 1: Fig. S3b).

The composition of the cellulolytic community and the whole microbial community was similar in both the metagenome and metatranscriptome (Fig. 2). At transcript level, the expression of cellulase genes at the T1 phase was significantly higher than that at the T2 phase (Fig. 2d), which might be related to the high CMCase activity at T1 phase. Furthermore, the expression of cellulase genes in treatments with high glucose (G_H_C_H_ and G_H_C_L_) was lower than CK and the expression of cellulase genes in treatment C_L_ was significantly higher than CK at the T2 phase. A suitable concentration of cellobiose that could induce cellulase production has been reported [35]. The genes involved in cellulose degradation, including endoglucanase, exoglucanase and β-glucosidase, were detected in the metagenome and metatranscriptome (Additional file 1: Fig. S4). The metagenome and metatranscriptome profiles of the key enzyme microbiome are similarly to the cellulolytic microbial community. At the DNA level, the abundance of β-glucosidase genes from the GH3 family in all treatments was higher than the GH1 family at T1 and T2 phases. However, at the transcript level, except for CK at T2 phase, the expression of β-glucosidase genes from the GH1 family in all treatments was higher than the GH3 family. This result corroborated the observation of Simmons [36] that GH1 and GH3 family genes associated with lignocellulolytic activity overexpressed in thermophilic and mesophilic communities, respectively. Moreover, the expression of β-glucosidase genes in C_L_ was higher, while it was lower in G_H_C_H_ compared with other treatments.

**Fig. 3:**
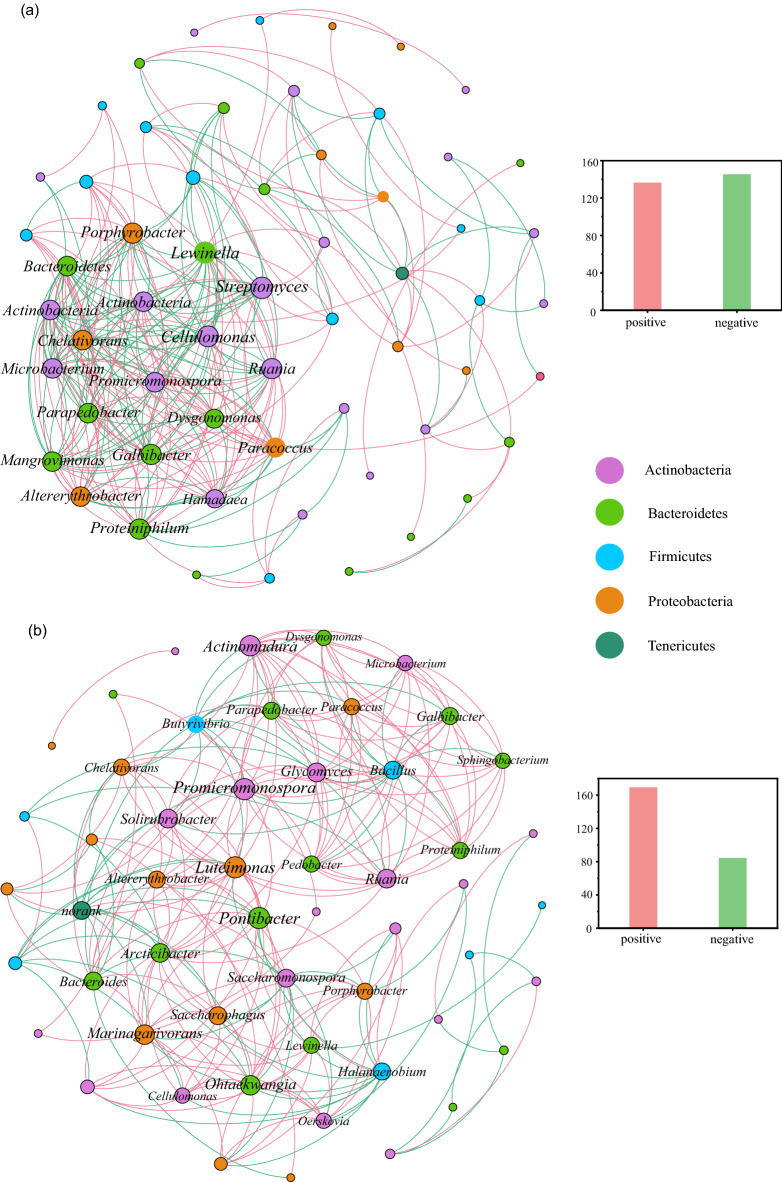
Network analysis of the β-glucosidase-producing microbial community co-occurrence patterns in the high-glucose (**a**) and non-glucose (**b**) treatments during composting in metagenome. The size of the nodes is proportional to the number of connections. Green lines denote positive linear relationships and red lines represent negative linear relationships

We constructed two networks to detect the intra-interactions of β-glucosidase-producing microorganisms in the high-glucose and non-glucose treatments in metagenome (Fig. 3). Network analysis revealed markedly different co-occurrence patterns between the glucose and non-glucose treatments. In general, the glucose treatments exhibited more highly interconnected cliques than non-glucose treatments. The co-occurrence network analyses revealed the majority of functional communities to be positively correlated in non-glucose treatments, whereas higher negatively correlated in high-glucose treatments, indicating that the carbon stress increases competition in the functional microbial communities. Notably, only part phyla of functional microorganisms (*Actinobacteria*, *Bacteroidetes*, and *Proteobacteria*) maintains the core network of high-glucose treatments. Hence, under the condition of carbon metabolic stress, the interaction of functional microbial communities will change due to the role of environmental screening and niche differentiation.

### Characterization of GH1 β-glucosidase‑producing microbial communities by DNA clone library

**Fig. 4:**
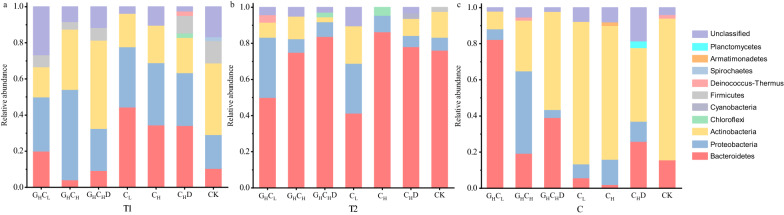
Relative taxonomic distribution of GH1 family bacterial β-glucosidase genes from the DNA library during composting procedures. Colors in stack bars indicate the taxonomic affiliation of genes. Unclassified sequences belong to bacteria, but their identification at the phylum level is unclear

Based on the observation that *Trp168* and *Leu173* were conserved in glucose-tolerant GH1 family β-glucosidase, the 1100–1200 bp GH1 β-glucosidase genes including the characteristic sequence was amplified, making it possible to identify the glucose tolerant or non-glucose tolerant β-glucosidase genes. This identification method has been carried out successfully by Zhang [37]. A total of 991 GH1 family bacterial β-glucosidase genes were obtained from the DNA clone library. The composition of the GH1 β-glucosidase-producing bacterial communities was analyzed in all treatments of compost at T1, T2 and C phases (Fig. 4). *Actinobacteria*, *Proteobacteria* and *Bacteroides* dominated the β-glucosidase producing bacterial communities across all samples at the phylum level, which is consistent with Pathan et al. [13]. Meanwhile, *Actinobacteria*, *Proteobacteria* and *Bacteroidetes* showed regular changes at different phases and treatments during composting. *Proteobacteria* were registered to be dominant at the T1 phase, but their abundance decreased as composting progressed. *Actinobacteria* were dominant during the T1 and C phases, while *Bacteroides* were dominant at the T2 phase.

**Fig. 5:**
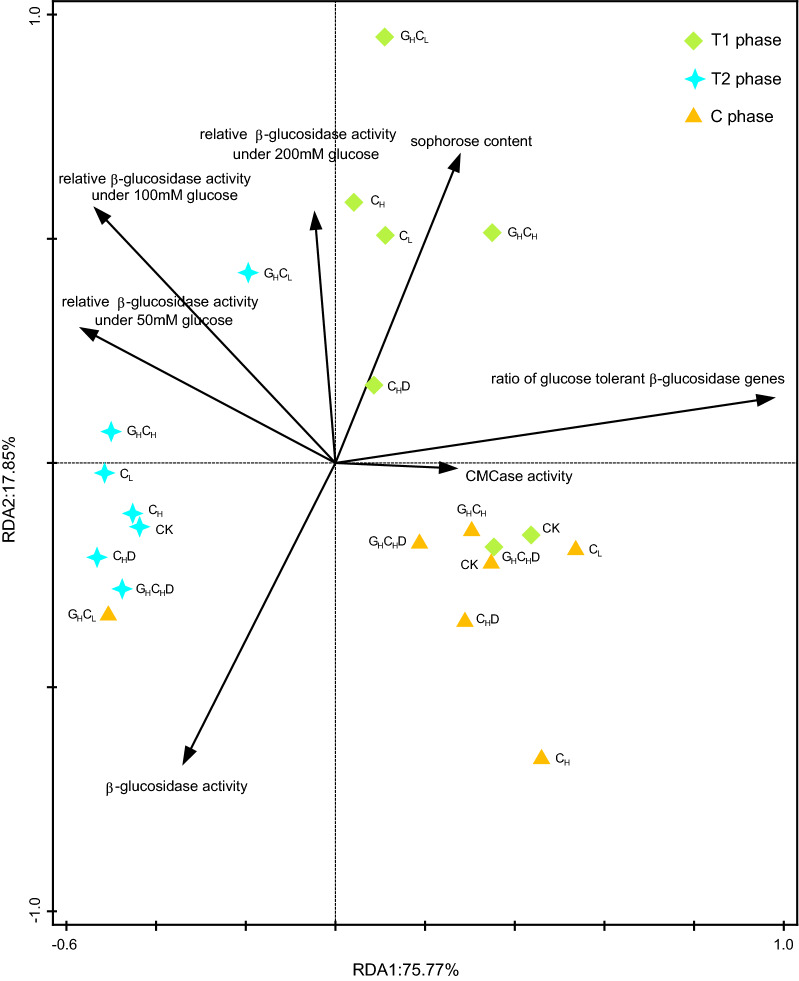
RDA analyses of the GH1 family β-glucosidase-producing microbial community composition on the class level within the different treatments from different phases and physicochemical parameters

The RDA profile showed the relationships among biotic and abiotic factors and GH1 family β-glucosidase community composition from the DNA clone library (Fig. 5). The first and second RDA components (RDA1 and RDA2) explained 93.62% of total GH1 family β-glucosidase community composition variations. The composition of the activated functional microbial community in treatment G_H_C_L_ at T2 and C phases (the main phase of cellulose degradation) were more resistant to environment perturbation of external carbon source addition than the T1 phase. Most treatments were most correlated with sophorose content at the T1 phase. Notably, G_H_C_L_ treatment was most correlated with the relative activity of β-glucosidase at the T2 phase.

The ratio of glucose-tolerant β-glucosidase genes and total β-glucosidase genes was calculated from the DNA clone library. The ratio of each treatment at the T2 phase was lower than that of the T1 and C phases. Interestingly, the ratio of treatment G_H_C_L_ was significantly higher than other treatments at the T2 phase (Additional file 2: Table S2). These results indicated that non-glucose β-glucosidase plays a vital role in the β-glucosidase activity, which changed in G_H_C_L_ treatment at the T2 phase. Furthermore, glucose-tolerant conservative residues were not found in all 381 β-glucosidase genes that belonged to *Bacteroides*. In contrast, approximately 80% of β-glucosidase genes belonged to *Actinobacteria* had glucose-tolerant conservative residues.

### Differential expression of glucose tolerance and non-glucose tolerance β-glucosidase genes

**Fig. 6:**
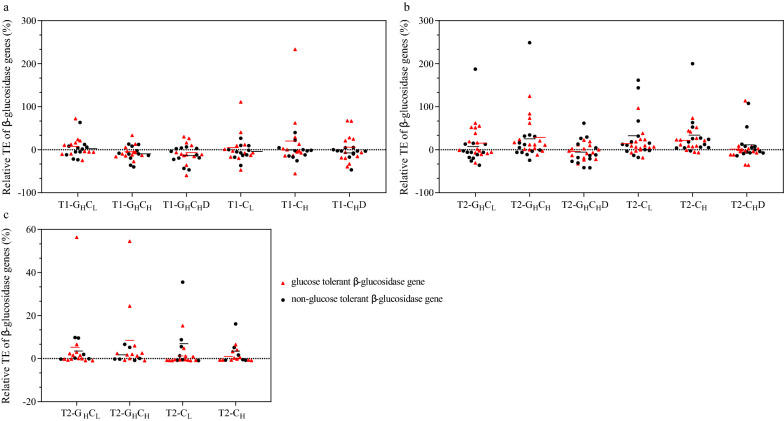
Relative transcription efficiency of individual β-glucosidase genes during composting procedures. **a** Relative transcription efficiency of individual β-glucosidase genes at the T1 phase using the qPCR method. **b** Relative transcription efficiency of individual β-glucosidase genes at T2 phase using qPCR method. **c** Relative transcription efficiency of individual β-glucosidase genes at T2 phase using metagenome and metatranscriptome. The red line represents the average relative transcription efficiency of glucose tolerant β-glucosidase genes. The black line represents the average relative transcription efficiency of non-glucose tolerant β-glucosidase genes

To explore the mechanism underlying the transcriptional regulation of β-glucosidase genes expression in the microbial community of different treatments at thermophilic phases (T1 and T2). The 31 representative β-glucosidase genes were selected from diverse bacterial phyla from the DNA library and quantitatively analyzed these genes from DNA and RNA pools (Additional file 2: Tables S3–S5). 17 of the 31 β-glucosidases were identified as glucose-tolerant β-glucosidase genes. For a better understanding of the expression level of the individual β-glucosidase gene, the TE and the relative TE were calculated (Fig. 6, Additional file 1: Fig. S2). The results of TE showed that the average TE of the glucose tolerant β-glucosidase gene is higher than the average TE of the non-glucose tolerant β-glucosidase gene in all treatments at the T1 phase (Additional file 1: Fig. S2a). In contrast, the average TE of the non-glucose tolerant β-glucosidase gene is higher than the average TE of the glucose tolerant β-glucosidase gene in all treatments at T2 the phase (Additional file 1: Fig. S2b). The results of relative TE of all treatments at the thermophilic phase are shown in Fig. 6. Interestingly, the average relative TE of the glucose tolerant β-glucosidase gene was higher than the average relative TE of the non-glucose tolerant β-glucosidase gene in all treatments at T1 phase and treatments with high glucose at T2 phase. Still, the average relative TE of the non-glucose tolerant β-glucosidase gene was higher than the average relative TE of the glucose tolerant β-glucosidase gene in treatments without glucose at T2 phase. These results indicated that functional microbial communities up-regulated glucose tolerant β-glucosidase genes and down-regulated non-glucose tolerant β-glucosidase genes under CCR.

The relative TE of individual β-glucosidase genes from metagenomic and metatranscriptomic were also calculated at T2 phase (Fig. 6c). 28 GH1 family β-glucosidase sequences, including function-discriminating residues (glucose or non-glucose tolerant), have been successfully mapped between metagenomic and metatranscriptomic data sets with ≥ 95% similarity. In the treatment with high glucose (G_H_C_L_ and G_H_C_H_) (Additional file 2: Table S6), the average relative TE of glucose-tolerant β-glucosidase genes was higher than non-glucose-tolerant β-glucosidase genes. In contrast, in the treatment without glucose (C_L_ and C_H_), the average relative TE of non-glucose tolerant β-glucosidase genes was higher than glucose tolerant β-glucosidase genes (Fig. 6c). These results further demonstrate that functional microbial community differential expresses glucose/non-glucose β-glucosidase genes under high glucose.

## Discussion

### The function and composition of the microbial community under CCR

The composition and function of the whole and cellulolytic microbial community were determined by metagenome and metatranscriptome. Previous research has shown that change in microbial community composition during composting is driven by alterations in the environmental conditions during each phase [38, 39]. In line with this concept, metagenome cluster analysis revealed that the change of microbial community composition due to environmental conditions differ from the composting phase. However, metatranscriptome cluster analysis revealed that the functional microbial community was more sensitive to short-term carbon metabolism pressure perturbation, especially in the G_H_C_H_ treatment (Additional file 1: Fig. S3). G_H_C_H_ treatment produced a lot of simple carbon sources for microbes and exhibited the CCR effect. The functional microbial community undergoes a series of changes under the G_H_C_H_ treatment. During the active phase of cellulose degradation (T2 phase), the abundance of carbohydrate transport and metabolism genes, GH genes and β-glucosidase genes were increased in the G_H_C_H_ treatment. However, the expression of these genes was decreased. These results indicated that the CCR effect inhibited carbohydrate metabolism (especially cellulose degradation). In G_H_C_H_ treatment, the microbial community composition shifted; the abundance of *Firmicutes* increased, while *Actinobacteria* decreased. Considering that community structure is closely related to the function of microbial communities [7]. *Actinobacteria* were known to play an important role in decomposing organic materials in composts [34, 37]. The observed *Actinobacteria* decrease in G_H_C_H_ treatment might be due to self-adaptation mechanism of functional microbes under CCR. It was also observed that the abundance of *Actinobacteria* decreased under high glucose in the DNA library dominated by *Actinobacteria* at the C phase.

To focus on smaller scale functional microbial communities and how they respond to different carbon metabolism pressures, the networks to detect the intra-interactions of β-glucosidase-producing microorganisms in the high-glucose and non-glucose treatments were constructed in metagenome, and GH1 family β-glucosidase-producing bacterial communities were analyzed by constructing a DNA library. Microbial co-occurrence networks showed marked differences between the high-glucose and non-glucose treatments. Negative correlations in taxa co-occurrence possibly indicate taxa competition [40]. In this study, network results showed that more negative connections were found in high glucose treatments (Fig. 3). Meanwhile, compared with the non-glucose treatments, the functional microbial co-occurrence network in high glucose treatments exhibited more highly clustered. These results implied that high glucose caused strong environmental selection on functional microbial communities. Furthermore, the DNA library results showed that the *Bacteroidetes* and *Actinobacteria* were the predominant phylum of β-glucosidase-producing bacterial communities in the T2 and C phases, respectively (Fig. 4c, d). As mentioned above, the changes in microbial community composition would affect the function of microbial communities. Cellulolytic-producing microbes can be divided into two groups: degraders (contain genes for both cellulases and β-glucosidases) and opportunists (contain genes encoding β-glucosidases but no cellulases) [41, 42]. A recent analysis of 5123 annotated bacterial genomes revealed that only 24% are classified as potential cellulose degraders, while 56% are classified as potential opportunists [12]. Fierer et al. [43] pointed out that opportunists appear to dominate in *Bacteroidetes* and the potential degraders were common in *Actinobacteria*, *Firmicutes* and *Proteobacteria* [12]. In this study, the abundance of *Bacteroidetes* increased considerably at the T2 phase from the DNA clone library. These results indicated that more opportunists participated in cellulose degradation at the T2 phase, which accelerated the degradation of cellulose. As cellulose degraded slowly during the C phase, cellulose degraders indicated by *Actinobacteria* were preponderant, while opportunists indicated by *Bacteroidetes* retreated.

Furthermore, different from CMCase, the activity of β-glucosidase was not affected by high glucose concentration at T1, T2 and C phases during composting (Fig. 1a, b). A similar observation has been documented in previous studies [44, 45], in which the activity of β-glucosidase showed insensitivity to environmental variations compared with endoglucanase and exoglucanase, because of the wider phylogenetic diversity of microorganisms capable of expressing β-glucosidases [46]. The β-glucosidase-producing microbial communities harboring a high degree of functional redundancy and thus better buffered against environmental disturbance.

The result of RDA analyses of microbial community targeted by β-glucosidase genes from DNA clone library and physicochemical parameters showed that functional microbial community composition of T2 and C (the main phase of cellulose degradation) exhibited stability in response to environment disturbance (Fig. 5). The DGGE profile of Li’s study [30] showed that the β-glucosidase producing microbial community composition in the T2 phase was more stable than that in other phases. T2 and C were the main phases of cellulose degradation. The composition of the functional microbial community was stable and their functions were activated after environmental selection and competition. Thus, the functional microbial communities were more resistant to environmental disturbances.

Interestingly, the obvious change in the composition and function of functional microbial community was observed in the G_H_C_L_ treatment. The functional microbial community differential regulated glucose/non-glucose tolerant β-glucosidase expression in the G_H_C_L_ treatment. Unlike G_H_C_H_ treatment that causes obvious changes in the composition and function of the microbial community on a larger scale (broad functions), G_H_C_L_ treatment had a stronger effect on the changes in the GH1 family β-glucosidase-producing microbial community within a small-scale (narrow function). These results indicated that microbial communities at different scales exhibited different responses to environmental disturbance. Compared with the broad functions of microbial communities, the microbial communities can be defined with respect to narrow functions that were more sensitive to environmental disturbance and tightly regulated under stress.

### Functional microorganism differential express β-glucosidases at thermophilic phase (T1 and T2) of compost

The CCR is caused by high glucose accumulation during the composting process, which impaired cellulose degradation by inhibiting the expression and hydrolysis activity of cellulase. The T1 phase, the early thermophilic stage of composting, has many simple carbon compounds and is widely known, such as polysaccharides and starch [30]. Cellulose as recalcitrant biopolymers, which decomposition was inhibited at the T1 phase. β-Glucosidase is considered a rate-limiting enzyme during the cellulose degradation process [9, 10, 47], the hydrolysis activity of β-glucosidase at T1 phase lower than T2 and C phases (Fig. 1b). Considering CMCase activity kept a high level at the T1 phase, indicating that the cellulose degradation at the T1 phase was limited to the step that β-glucosidase hydrolyzes cellobiose to glucose.

The CMCase activity and the expression of cellulolytic genes kept at a high level at the T1 phase, which might be due to the wild existence of sophorose at the T1 phase. Sophorose regarded as a potent inducer of cellulase expression, has been reported [31]. Sophorose is the product of the transglycosylation of β-glucosidase [48, 49]. Transglycosylation of β-glucosidase occurred in the T1 phase, and the activity increased under high glucose treatments. We speculate that functional microbial communities respond to environmental changes by adjusting β-glucosidase hydrolysis and transglycosylation activities, which also show at the transcriptional level. TE analysis of individual GH1 family β-glucosidase genes from the clone library by qRT-PCR, functional microbial community down-regulated expression of non-glucose tolerant β-glucosidase that contributes mainly to the key β-glucosidase hydrolysis activity, could explain why the β-glucosidase activity kept at low level at the T1 phase. Meanwhile, functional microbial community up-regulated expression of glucose tolerant β-glucosidase, which was related to the high level of transglycosylation activity of β-glucosidase at the T1 phase. In some reports, some β-glucosidases with transglycosylation activity exhibited glucose tolerance [50, 51]. The phenomenon that the hydrolytic activity was compensated by increased transglycosylation was also found in the study of Bohlin [52]. To respond to the effect of environmental change on process rate, the functional microbial community alters the metabolic pathways via differential expression glucose/non-glucose tolerant β-glucosidase genes. The differential expression of functional microbial community occurred under the CCR background of the T1 phase. In addition, the addition of easily degraded carbon sources (glucose and cellobiose) can enhance this differential expression under CCR.

The cellulase gene was regulated by glucose and cellobiose [7, 35]. Previous research reported that although cellobiose is considered a poor inducer [31], cellulase expression was induced by suitable concentration cellobiose and inhibited by high concentration cellobiose [35]. In the T2 phase, the expression of cellulolytic genes and CMCase activity were inhibited by high concentration glucose (G_H_C_L_, G_H_C_H_), but the expression of cellulolytic genes was induced by low concentration cellobiose (C_L_). As expected, this induction did not occur in treatment C_H_.

Different from the T1 phase with a CCR background, cellulose was degraded rapidly at the T2 phase. The qRT-PCR result of individual GH1 family β-glucosidase genes showed that the average TE of non-glucose tolerant β-glucosidase genes was higher than glucose tolerant β-glucosidase genes in all treatments at the T2 phase. The difference of average TE between glucose tolerant and non-glucose tolerant β-glucosidase genes in treatments with high glucose (G_H_C_H_, G_H_C_L_, G_H_C_H_D) was lower than that in other treatments. These results indicated that under the general condition that no CCR effect occurs during cellulose degradation, the functional microbial communities mainly expressed non-glucose tolerant β-glucosidase participate in the process of cellulose degradation. This result was consistent with Zhang [7] and Mathew [53], which is a prevalent economic microbial behavior. Meanwhile, the ratio of glucose-tolerant β-glucosidase genes in all treatments at T2 phase was lower than that at the T1 phase in the DNA library (Additional file 2: Table S2), indicating that the non-glucose β-glucosidase genes are the main components of β-glucosidase genes at T2 phase.

The relative TE was used to describe the effects of different carbon metabolic pressure on transcriptional regulation of β-glucosidase genes. The average relative TE of glucose tolerant β-glucosidase genes was higher than non-glucose tolerant β-glucosidase genes in treatments of high glucose at the T2 phase (Fig. 6b, c), but it was the opposite in the treatments without glucose. The result showed that the functional community could regulate the expression of glucose/non-glucose tolerant β-glucosidases to respond to the CCR caused by high glucose [7]. To overcome the limitation of the constructed DNA library method, the combined analysis of metagenome and metatranscription was used in this study. The results obtained by these methods are consistent, indicating the differential expression of the functional microbial community is a widespread response mechanism rather than random events. Meanwhile, the ratio of glucose-tolerant β-glucosidase genes expression in treatments with high glucose (89.42% and 89.18%) was higher than other treatments (64–72%) (Additional file 2: Table S7). These results present evidence of β-glucosidase genes differential expression under high glucose. Sophorose was detected in high glucose treatments, which occurred under β-glucosidase genes differential expression. This differential expression is related to the enhancement of the transglycosylation activity of β-glucosidase to compensate for the decrease in hydrolysis activity. For the decisive role of fungi in the C phase, the function of the target microbial community is weakened, cause regulatory behavior of target microbial community differential expression was not obvious in this phase (unpublished).

**Fig. 7:**
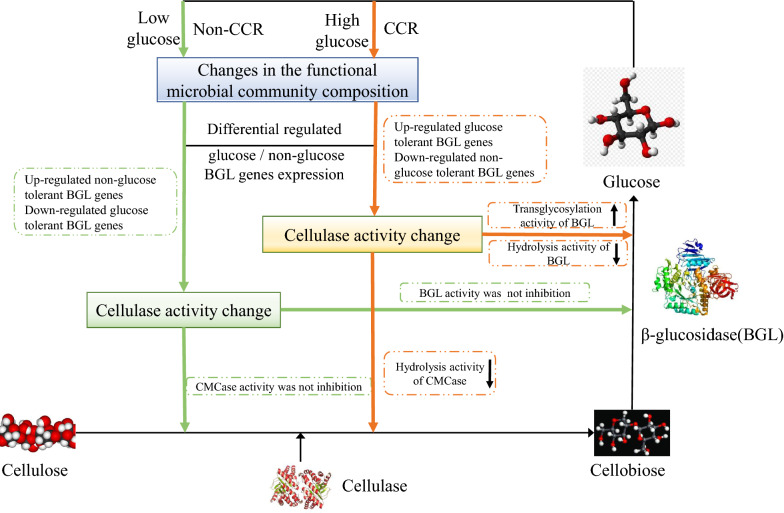
Schematic diagram of expression regulation of functional microbial community in the cellulose degradation process. Orange lines and arrows represent expression regulation of functional microbial community under CCR. Green lines and arrows represent expression regulation of functional microbial community under non-CCR. The arrows in the dashed box represent the increase and decrease of enzyme activity

Based on the previous studies from our team [7, 16, 17, 30], and this current study. We found that the functional microbial communities via differential regulated expression of glucose/non-glucose tolerant β-glucosidase genes, respond to the change of glucose content (CCR or non-CCR environment) during cellulose degradation. These regulatory processes are shown in Fig. 7.

### Functional microbe response behavior at the phylogenetic level

Further analysis of the phylogeny of GH1 family glucose/non-glucose tolerant β-glucosidase genes showed that the differentially expressed β-glucosidases seem to be related to certain populations. The proportion of glucose-tolerant β-glucosidase genes belonging to *Actinobacteria* is 80% in the DNA library, but the β-glucosidase genes from Bacteroides that dominate at T2 the phase are non-glucose tolerant β-glucosidase genes. These results indicated that the differentially expressed β-glucosidase gene exhibited species characteristics at the phylogenetic level. Interestingly, 14 GH1 family β-glucosidase genes from the *Micrococcales* were detected in metagenome and metatranscriptome databases that can identify glucose tolerance and non-glucose tolerance, of which 13 were glucose tolerant β-glucosidase genes (Additional file 2: Tables S7, S8). Among the 22 β-glucosidase genes calculated for relative TE, two genes belonging to *Micrococcales*, were overexpressed in high glucose treatment. Meanwhile, in our previous study, the glucose tolerant β-glucosidase genes GH1B-7-4-17, GH1B-13-55 and GH1B-12-33 belonged to *Micrococcales*, the expression of GH1B-13-55 and GH1B-12-33 genes were up-regulated in the cooling phase of natural compost which CCR occurred [7]. These results indicated that *Micrococcales* plays an important role in the resistance of functional microbial community under CCR. We speculated that the up-regulated glucose tolerant β-glucosidases could induce cellulase formation by sophorose produced by transglycosylation [54, 55]. In Ríos-Fránquez’s [54] study, *Cellulomonas flavigena* PR-22 showed more resistance to catabolic repression than its predecessor PN-120 strain, and the β-glucosidase produced by PR-22 exhibited more glucose tolerance. Hiida Rodriguez et al. [55] reported that β-glucosidase could play a role in the formation of a natural cellulase inducer during the growth of *Cellulomonas* on sugarcane bagasse [55] we speculated that the cellulase inducer was a transglycosylation product of β-glucosidase.

In the treatment G_H_C_L_, the transcription regulation of GH1 family β-glucosidase genes from Proteobacteria showed more obvious inhibition than *Actinobacteria*, *Bacteroidetes* and *Firmicutes* (Additional file 1: Fig. S5). These results indicated that functional microorganisms of different classifications have different sensitivity to carbon metabolic pressure.

## Conclusion

Microbial functional communities differentially regulate the expression of glucose/non-glucose tolerant β-glucosidase genes under CCR, which is a general regulatory mechanism, not accidental. Functional microbial community up-regulated glucose tolerant β-glucosidase genes expression to maintain the carbon metabolism rate by enhancing the transglycosylation activity of β-glucosidase, thereby compensating for the decrease hydrolysis activity under CCR. Furthermore, the differentially expressed β-glucosidase gene exhibited species characteristics at the phylogenetic level.

## Materials and methods

### Composting construction and samples

Aerobic composting of cattle manure-corn straw was carried out at the Experimental Farm of Northeast Agricultural University, Acheng city, Harbin, China. The raw materials (cattle manure and corn straw) were homogenously mixed at a ratio of 5:1 (wt/wt) on dry weight basis. At the beginning of composting, the moisture content was adjusted to 60%. The pile was sub-divided into three smaller piles of 2.5 m × 1.5 m × 1 m (length × width × height). The piles were turned manually three times (days 11, 20 and 29) during the process to enable uniform degradation and airflow in the composting matrix and reduced compaction. Moisture content in the matrix was maintained between 40 and 60% by adding water periodically. The temperature of the piles was monitored every day using digital thermometer (XMD-110, Hengshui, China).

Samples were collected at day 16 (early thermophilic phase denoted as T1 phase), day 23 (later thermophilic phase denoted as T2 phase) and day 35 (cooling phase denoted as C phase) and sampling strategy was designed to ensure a representative sample of the piles. In total, 27 sub-samples were taken from nine different locations of each pile as follows: 3 samples at 15 cm depth, 3 samples at 50 cm depth and 3 samples at 90 cm depth. Samples of the same piles were mixed in equal amounts to give a final sample weight of about 5.6 kg and then split into seven parts of about 0.8 kg. The seven parts were sprayed with their respective treatment solutions (40 mL), then each of the treated parts were divided into three equal parts and returned to the center of the three first made piles for analytical replicates. After 6 h of incubation, samples were taken, kept in liquid nitrogen during transportation and thereafter at − 80 °C in the laboratory. The final additive incubation concentrations and treatments name used are showed in Table 1. The eight samples (T1-G_H_C_H_, C_H_, CK and T2-C_H_C_L_, G_H_C_H_, C_L_, C_H_, CK) were used for metagenomics and metatranscriptomics analyses.

**Table 1:** Additive incubation concentrations for each treatment

	G_H_C_L_	G_H_C_H_	G_H_C_H_D	C_L_	C_H_	C_H_D	CK
Glucose (mmol/L)	200	200	200	0	0	0	0
Cellobiose (mmol/L)	0.8	8	8	0.8	8	8	0
d-Glucono-1,5-lactone (mmol/L)	0	0	2	0	0	2	0

### Enzymatic activities, content of glucose, cellobiose and sophorose analyses

The assay of CMCase activity was estimated following the procedure described by Zang et al. [16]. The activity of β-glucosidase was measured using *p*-nitrophenyl β-d-glucoside (PNPG) as described by Zang et al. [16]. Relative activities of β-glucosidase under gradient glucose concentration were determined by a glucose tolerant test. The concentration of the added glucose solution was 50, 100 and 200 mmol/L, respectively.

The glucose, cellobiose and sophorose contents in the samples were determined with high-performance liquid chromatography (HPLC) using the Cosmosil-Sugar-D chromatographic column (Nacalai Tesque Inc., Japan) with 75% acetonitrile as the mobile phase, a column temperature of 60 °C, and a velocity of 0.5 mL/min, as assessed by 2414 index detector.

### Metagenomics and metatranscriptomic analysis

Total DNA was extracted using the E.Z.N.A.® stool DNA Kit (Omega Bio-tek, Norcross, GA, U.S.) according to manufacturer’s protocols. RNA contamination was removed using RNAase. DNA quality and quantity were determined using 2100 Bioanalyser (Agilent) and ND-2000 (NanoDrop Technologies), respectively. Metagenomic shotgun sequencing libraries were constructed, 1 μg of genomic DNA was sheared by Covaris S220 Focused-ultrasonicator (Woburn, MA USA) and sequencing libraries were prepared with a fragment length of approximately 450 bp. All samples were sequenced in the Illumina HiSeq 2500 instrument with pair-end 150 bp (PE150) mode.

Total RNA was extracted using TRIzol® Reagent according the manufacturer’s instructions (Invitrogen) and genomic DNA was removed using DNase I (TaKara). Then RNA quality was determined using 2100 Bioanalyser (Agilent) and quantified using the ND-2000 (NanoDrop Technologies). The details of metatranscriptome were described by Zhang [7].

Raw sequence reads of Metagenomics and metatranscriptomics sequencing underwent quality trimming using Trimmomatic to remove adaptor contaminants and low-quality reads [56]. The reads removing host-genome contaminations and low-quality data were called as clean reads and used for the further analysis.

Comparing metagenomic and metatranscriptomic data sets may reveal the relative activity levels of different populations in functional microbial community. Relative abundance of β-glucosidase in DNA and RNA levels was calculated using the abundant of individual glucosidase divided by the sum of all abundance of β-glucosidase genes with a characterized sequence that can identify glucose/non-glucose tolerant β-glucosidase genes. These relative abundance values were used to estimate transcription efficiency (TE, DNA/RNA ratio). The relative TE (Eq. 1) was used to describe the effects of different carbon metabolic pressure on transcriptional regulation of β-glucosidase genes. The average relative TE was used to describe the transcriptional regulation trend of the groups of glucose and non-glucose β-glucosidase genes. Equation (1) shows a mathematical model of relative TE of individual β-glucosidase genes in different treatments:

1 $${\text{relative}}\;{\text{TE }} = \frac{{{\text{TE}}_{{{\text{treatment}}}} - {\text{TE}}_{{{\text{ck}}}} }}{{{\text{TE}}_{{{\text{ck}}}} }},$$

where TE_treatment_ denotes the transcription efficiency of β-glucosidase gene in different treatments, and TE_ck_ is the transcription efficiency of β-glucosidase gene in CK.

### Annotation of the metagenome and metatranscriptome

All genes were compared against the NCBI protein non-redundant (NR), String and KEGG databases using BLASTp (Version 2.8.1+, http://blast.ncbi.nlm.nih.gov/Blast.cgi) to identify proteins with the highest sequence similarities provided unigenes to retrieve their function annotations, with an *E* value cutoff less than 1.0 × 10^−5^. Annotating the functional groups was done using the CAZy database (https://www.CAZy.org). Glycoside hydrolases (GH) and auxiliary enzyme (AA) families were grouped based on which substrate they act upon, as described in the study by Žifčáková [45].

### Construction of functional gene library

The detail of DNA and cDNA prepare was described by our previous research [7]. Clone libraries targeting the GH1 β-glucosidase genes from bacteria in all samples were constructed for studying functional communities in each sample. The detailed information on primer and reaction conditions of PCR was described by Zhang [7]. PCR products were separated by 1% electrophoresis and gels purified using Gel Extraction kit (Omega, Inc., USA). The purified products were ligated to pMD™18-T Vector (Takara, Japan), then transformed into *E. coil* DH5α competent cells using the heat shock method. The competent cells were incubated on solid Luria–Bertani (LB) medium containing X-gal, IPTG and ampicillin (AMP^+^) at 37 °C for 14 h. After which the white single colonies were used as PCR template to detect successful transformation in which positive strain containing the gene fragment were sent to Beijing Huada Gene Company (Beijing, PR China) for sequencing.

### Real-time qPCR of β-glucosidase genes

Thirty-one representative β-glucosidase genes were selected from the functional genes clone library across different bacterial phyla and were measured by quantification. Gene-specific primers were designed using the Primer 5.0 software and the Primer-BLAST in NCBI (National Center for Biotechnology Information). The gene-specific primers sequences as well as the GenBank accession numbers for the representative β-glucosidase gene sequences are shown in Additional file 2: Table S1.

The primers were designed based on sequences in the functional gene library (Additional file 2: Table S1). DNA and cDNA served as templates for the quantitative analysis of the β-glucosidase genes. The detailed information on reaction conditions real-time qPCR was described by Zhang et al. [7]. Real-time qPCR quantification of β-glucosidase genes was performed in triplicate as described in Additional file 2: Tables S3–S5.

The transcription efficiency (TE) of β-glucosidase genes was calculated using the ratios of both cDNA and DNA copy numbers. The relative TE of individual β-glucosidase genes in different treatments was described in Eq. (1).

### Statistical analysis

SPSS 22 and JMP 13 for windows were used for the statistical analysis. One-way repeated measures ANOVA was used to test differences in the measured parameters during composting, and post hoc Tukey’s test was used to further investigate these differences (*P* < 0.05). DEGseq of R package was used to identify differentially expressed genes. Co-occurrence network analyses were finished on the molecular ecological network analysis pipeline platform (MENA, http://ieg2.ou.edu/MENA/) [57], and visualized through the network graphs using Gephi following [58]. GraphPad Prism 8 and Origin 2019b were used for the data analysis and drawing data charts.

## Supplementary Information

**Additional file 1: Figure S1.** Changes in temperature of the aerobic composting pile. **Figure S2.** Transcription efficiency of individual β-glucosidase genes in **a** T1 and **b** T2 phase of compost using qPCR method. **Figure S3.** Different treatments and different phases of composting samples microbial community at phylum level Heatmap in **a** metagenome and **b** metatranscriptome. **Figure S4.** Abundant and expression of key enzymes genes in cellulose degradation during composting. **Figure S5.** Relative transcription efficiency and phylogenetic analysis of β-glucosidase genes of treatments in T2 phase of compost using qPCR method.

**Additional file 2: Table S1.** Primers of β-glucosidase genes for qPCR. **Table S2.** The ratio of glucose-tolerant β-glucosidase genes of different treatments at T1, T2, and C phase from DNA library. **Table S3.** Results and statistics of qPCR and qRT-PCR of treatment groups in T1 phase. **Table S4.** Results and statistics of qPCR and qRT-PCR of treatment groups in T2 phase. **Table S5.** Results and statistics of qPCR and qRT-PCR of treatment groups in C phase. **Table S6.** Identity of 28 GH1 family β-glucosidase genes with a characteristic sequence that can identify glucose tolerant or non-glucose tolerant detected in both metagenome and metatranscriptome database. **Table S7.** GH1 family β-glucosidase genes with a characteristic sequence that can identify glucose tolerant or non-glucose tolerant detected in the metatranscriptome database. **Table S8.** GH1 family β-glucosidase genes with a characteristic sequence that can identify glucose tolerant or non-glucose tolerant detected in the metagenome database.

## Data Availability

All data generated or analyzed during this study are included in this published article.

## Abbreviations

GH: Glycoside hydrolases
AA: Auxiliary activities
CAZy: Carbohydrate-active enzymes
BGL: β-Glucosidase
pNPG: *p*-Nitrophenyl-β-d-glucopyranoside;
CCR: Carbon catabolite repression
CMC: Carboxymethyl cellulose
TPM: Transcripts Per Million
TE: Transcription efficiency
IPTG: Isopropyl-β-thiogalactopyranoside
