# Supplementary material for: Mechanism of differential expression of β-glucosidase genes in functional microbial communities in response to carbon catabolite repression
Source: Biotechnol Biofuels Bioprod. 2022 Jan 12;15:3. doi: 10.1186/s13068-021-02101-x (PMC8756671; doi:10.1186/s13068-021-02101-x)
Supplement: Supplementary file 1 — Additional file 1: Figure S1. Changes in temperature of the aerobic composting pile. Figure S2. Transcription efficiency of individual β-glucosidase genes in a T1 and b T2 phase of compost using qPCR method. Figure S3. Different treatments and different phases of composting samples microbial community at phylum level Heatmap in a metagenome and b metatranscriptome. Figure S4. Abundant and expression of key enzymes genes in cellulose degradation during composting. Figure S5. Relative transcription efficiency and phylogenetic analysis of β-glucosidase genes of treatments in T2 phase of compost using qPCR method. [file 13068_2021_2101_MOESM1_ESM.docx]

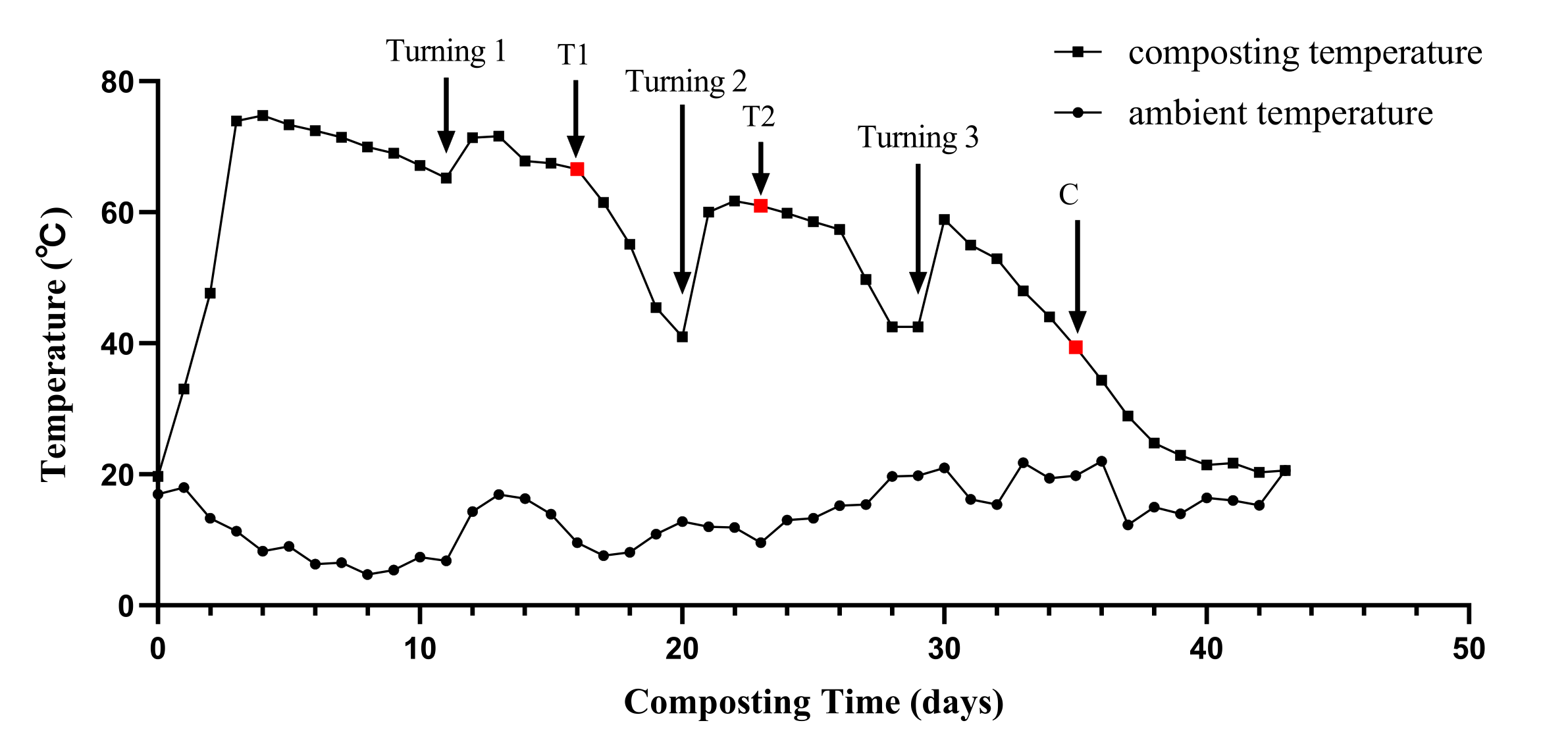


**Fig. S1 Changes in temperature of the aerobic composting pile.**


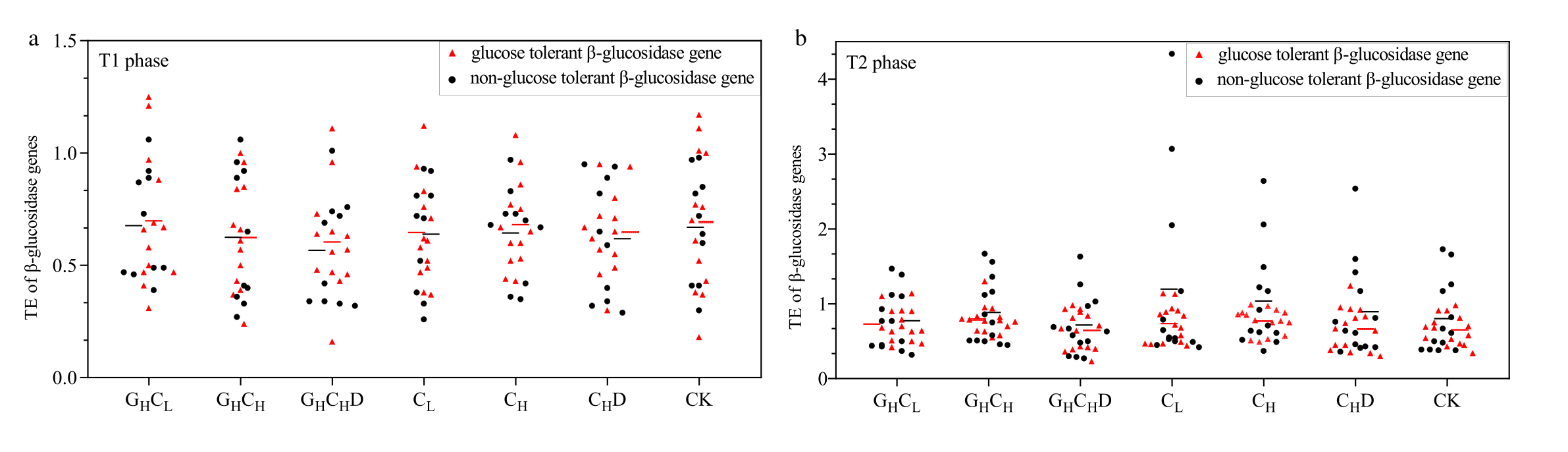


**Fig. S2 Transcription efficiency of individual β-glucosidase genes in a) T1 and b) T2 phase of compost using qPCR method.** The red line represents the average transcription efficiency of glucose tolerant β-glucosidase genes. The black line represents the average transcription efficiency of non-glucose tolerant β-glucosidase genes.


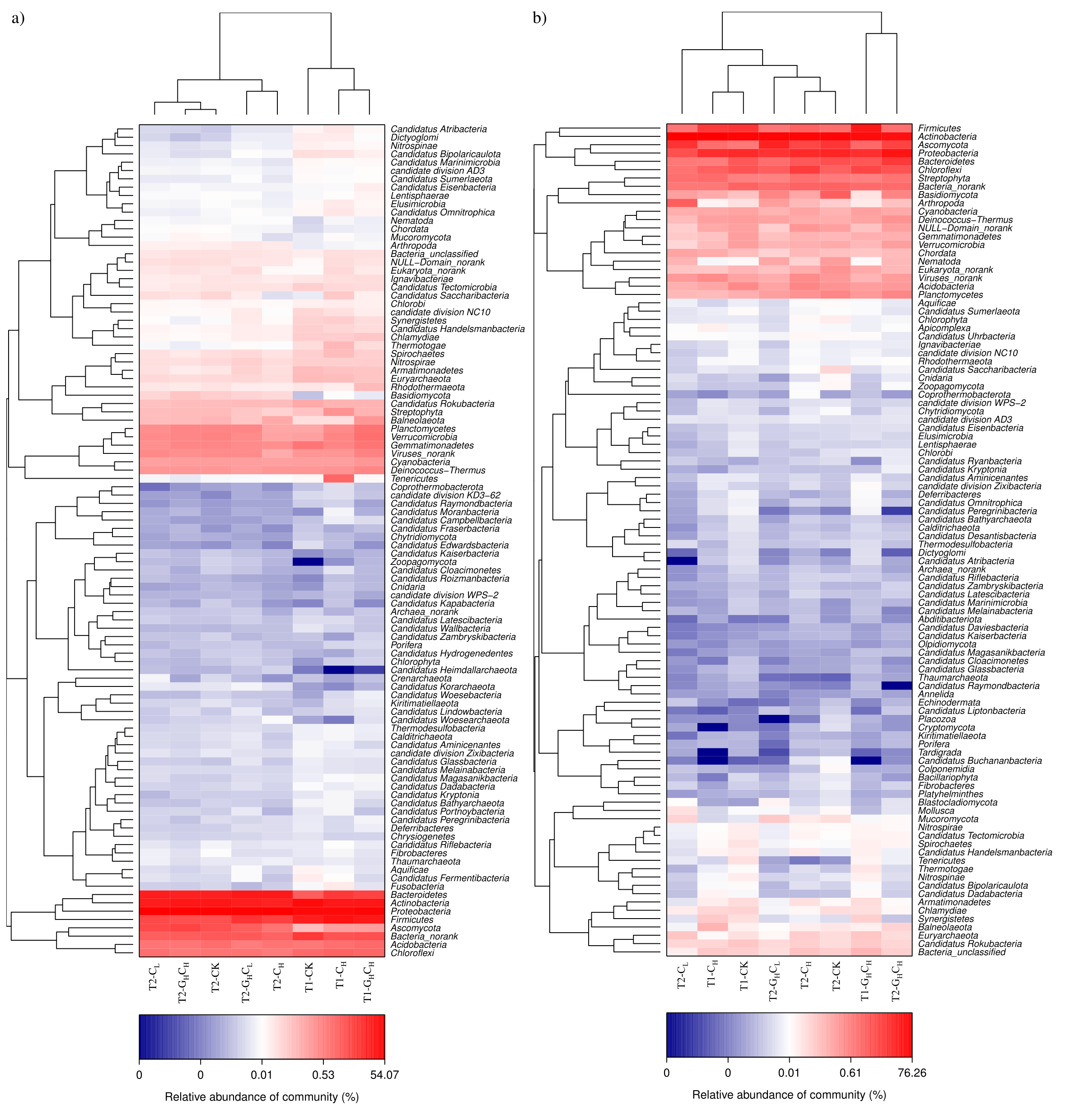


**Fig. S3 Different treatments and different phases of composting samples microbial community at phylum level Heatmap in a) metagenome and b) metatranscriptome.**


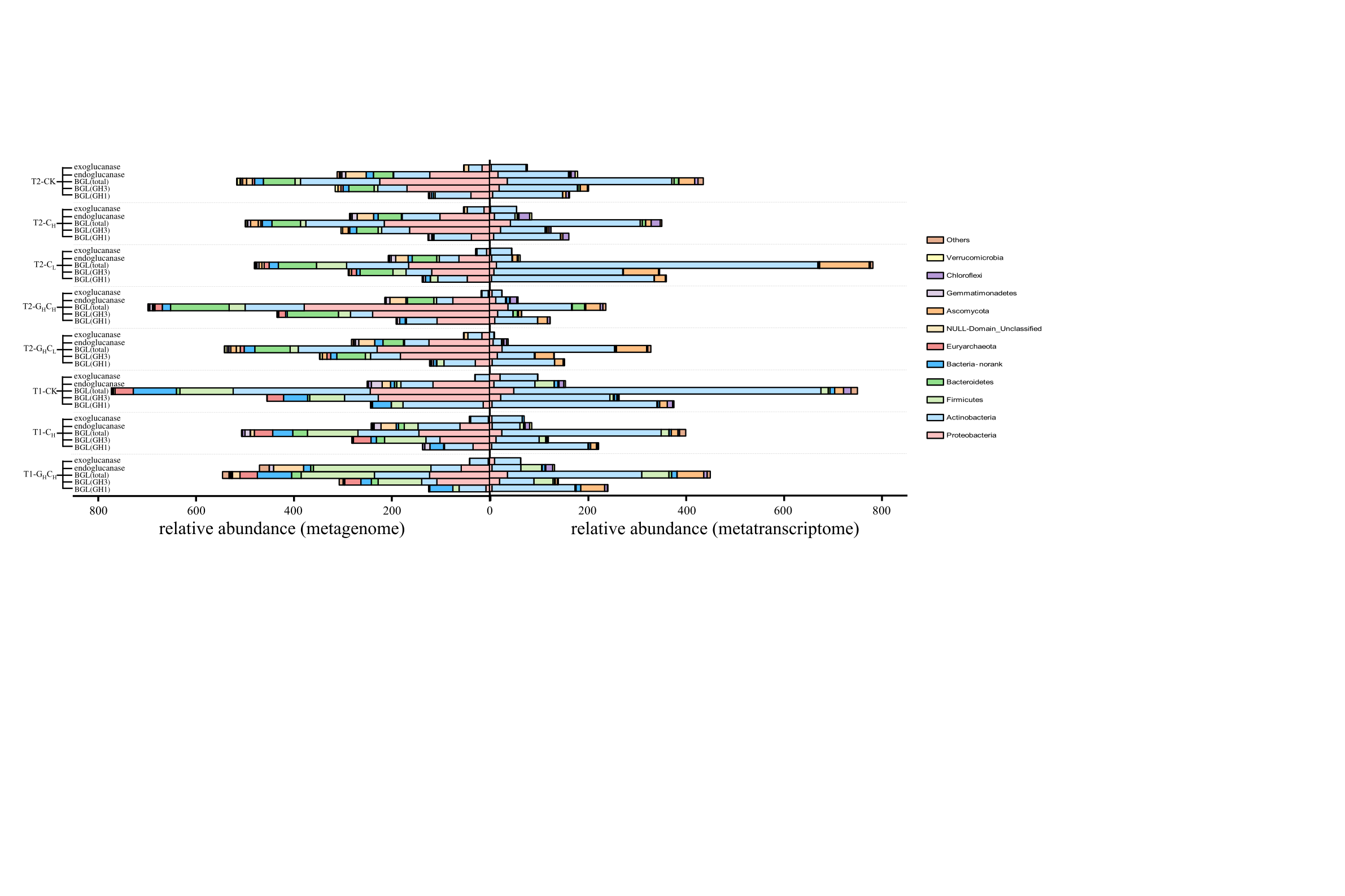


**Fig. S4 Abundant and expression of key enzymes genes in cellulose degradation during composting.** X-axis indicates the share of read in the total metagenome and metatranscriptome in TPM (Transcripts Per Million).


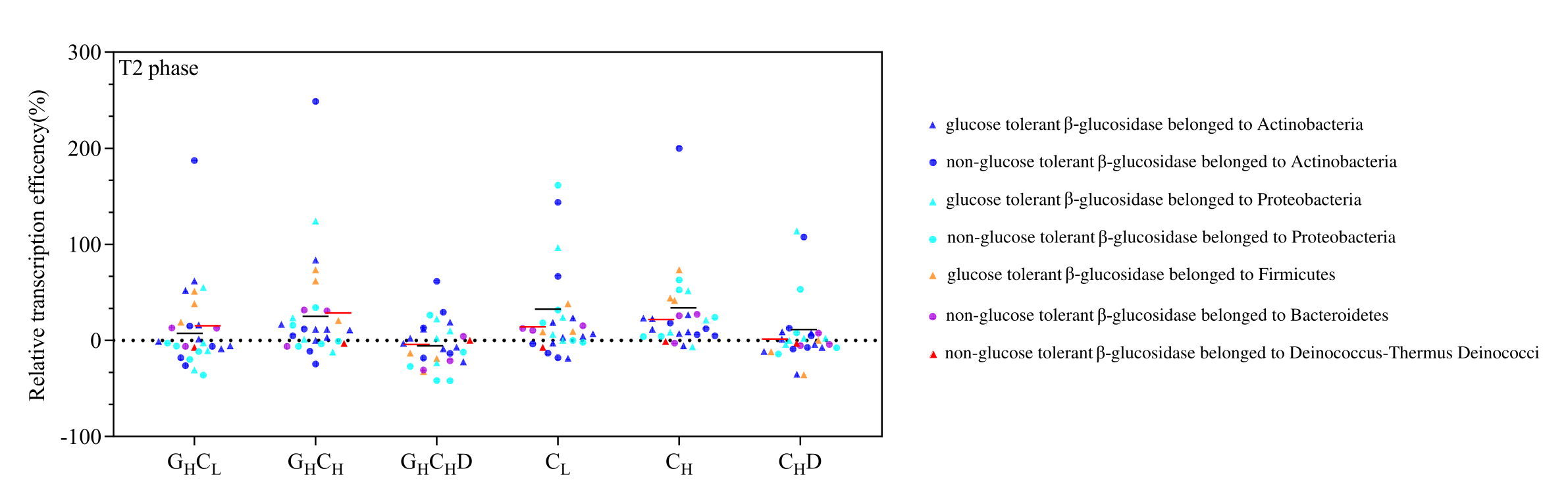


**Fig. S5 Relative transcription efficiency and phylogenetic analysis of β-glucosidase genes of treatments in T2 phase of compost using qPCR method.** The red line represents the average transcription efficiency of glucose tolerant β-glucosidase genes. The black line represents the average transcription efficiency of non-glucose tolerant β-glucosidase genes.
